# Genome-wide identification, phylogeny and expression analysis of AP2/ERF transcription factors family in sweet potato

**DOI:** 10.1186/s12864-021-08043-w

**Published:** 2021-10-16

**Authors:** Shutao He, Xiaomeng Hao, Shuli He, Xiaoge Hao, Peng Zhang, Xiaonan Chen

**Affiliations:** 1 State Key Laboratory of Cell Biology, Shanghai Institute of Biochemistry and Cell Biology, CAS Center for Excellence in Molecular Cell Science, Chinese Academy of Sciences, Shanghai, 200031 China; 2 National Key Laboratory of Plant Molecular Genetics, CAS Center for Excellence in Molecular Plant Sciences, Institute of Plant Physiology and Ecology, Chinese Academy of Sciences, Shanghai, 200032 China; 3 University of Chinese Academy of Sciences, Beijing, 100049 China; 4 Jining College Affiliated Senior High School, Jining, 272004 China; 5 Tsinghua University, Beijing, 100084 China; 6 Beihang University, Beijing, 100191 China

**Keywords:** Sweet potato, *IbAP2/ERF*, Development, Abiotic stress, Expression patterns

## Abstract

**Background:**

In recent years, much attention has been given to *AP2/ERF* transcription factors because they play indispensable roles in many biological processes, such as plant development and biotic and abiotic stress responses. Although *AP2/ERFs* have been thoroughly characterised in many plant species, the knowledge about this family in the sweet potato, which is a vital edible and medicinal crop, is still limited. In this study, a comprehensive genome-wide investigation was conducted to characterise the *AP2/ERF* gene family in the sweet potato.

**Results:**

Here, 198 *IbAP2/ERF* transcription factors were obtained. Phylogenetic analysis classified the members of the *IbAP2/ERF* family into three groups, namely, *ERF* (172 members), *AP2* (21 members) and *RAV* (5 members), which was consistent with the analysis of gene structure and conserved protein domains. The evolutionary characteristics of these *IbAP2/ERF* genes were systematically investigated by analysing chromosome location, conserved protein motifs and gene duplication events, indicating that the expansion of the *IbAP2/ERF* gene family may have been caused by tandem duplication. Furthermore, the analysis of *cis*-acting elements in *IbAP2/ERF* gene promoters implied that these genes may play crucial roles in plant growth, development and stress responses. Additionally, the available RNA-seq data and quantitative real-time PCR (qRT-PCR) were used to investigate the expression patterns of *IbAP2/ERF* genes during sweet potato root development as well as under multiple forms of abiotic stress, and we identified several developmental stage-specific and stress-responsive *IbAP2/ERF* genes. Furthermore, *g59127* was differentially expressed under various stress conditions and was identified as a nuclear protein, which was in line with predicted subcellular localization results.

**Conclusions:**

This study originally revealed the characteristics of the *IbAP2/ERF* superfamily and provides valuable resources for further evolutionary and functional investigations of *IbAP2/ERF* genes in the sweet potato.

**Supplementary Information:**

The online version contains supplementary material available at 10.1186/s12864-021-08043-w.

## Introduction

One of the largest gene families in plants is the *AP2/ERF* transcription factor (TF) superfamily, which includes at least one APETALA2 (AP2) domain comprising approximately 60 amino acid residues and is significant to the regulation of plant development and the response to various types of stress [1, 2]. By the rule of the components of conserved domains, *AP2/ERF* TFs can be separated into *ERF*, *AP2* and *RAV* gene subfamilies [3–5]. Most *AP2/ERF* TFs belong to the *ERF* subfamily, which contains one conserved AP2 domain, and the *AP2* subfamily encodes proteins with two AP2 domains. Additionally, with the exception of one single AP2 domain, the *RAV* subfamily also includes a B3 DNA binding domain that is conserved in other plant-specific transcription factors [6]. Although the sequences of the AP2 domain are highly conserved, the DNA binding elements of each subfamily are totally different. Generally, based on the DNA binding motifs, the *ERF* subfamily can be further subdivided into two groups: the *ERF* group, including B1 to B6 subgroups, can bind to the GCC-box (AGCCGCC element); and the *DREB* group, including A1 to A6 subgroups, can bind to the DRE/CRT (dehydration responsive element/C-repeat, RCCGCC element) element [7, 8].. The *AP2* subfamily cannot bind to the CCGA/CC element, which is a core element interacting with the *ERF* subfamily, but can bind to the GCAC(A/G)N(A/T)TCCC(A/G)ANG(C/T) sequence [9, 10]. Additionally, the binding sequences of the *RAV* subfamily can be CACCTG and CAACA elements [11].

Extensive studies have been conducted to investigate the significant roles of *AP2/ERF* genes in regulating diverse developmental processes, stress responses and plant hormone signal transduction, including ethylene, auxin, salicylic acid and jasmonic acid [12–14]. Several members of the *ERF* subfamily have been revealed as viable candidates to enhance plant abiotic stress tolerance and display different response patterns under abiotic stress, including cold (*AtCBF1*, *PtERF109*) [15, 16], heat (*ZmDREB2A*, *AtDREB1A*) [17, 18], osmotic stress (*CkDREB*) [19], drought (*OsDREB1*, *IbRAP2–12*) [20–22], and high-salt stress (*CaDREBLP1*, *LkERF-B2, IbERF5*) [23–25]. Members of the *AP2* subfamily play vital roles in the regulation of organ architecture and development, such as floral organ patterning [26–28], leaf development [29] and embryo development [30], while *RAV* subfamily genes are the main factors involved in plant hormone signal transduction [31], including that of brassinosteroids [32], ethylene [33] and auxin [34], and are the main regulators of multiple stress responses [11, 35, 36]. Thus, identification and analysis of the *AP2/ERF* gene family are crucial to understanding the mechanisms of many developmental processes and various stress responses.

The sweet potato [37], which originated in Central America and belongs to the Convolvulaceae family, is an important food crop grown globally and has significant medicinal value [38]. Additionally, with the release of sweet potato genome data and the advancement of transgenic technology, it has become possible to identify and investigate important gene families at the whole genome level. Beta-galactosidase family members of the sweet potato have been identified at the genome-wide level [37]. Additionally, genome-wide characterisations of several potassium absorption-related gene families, such as the HAK K^+^ transport family [39] and the Shaker K^+^ channel family [40], have also been investigated.

Due to the significance of *AP2/ERF* genes in many biological processes, it is crucial to systematically investigate the *AP2/ERF* gene family in the sweet potato. The functions of *AP2/ERF* TFs have been well studied in many species, but there are few investigations in the sweet potato. *IbDREB1* was identified and can respond to several abiotic stressors, including dehydration, salt and cold stress [41]. A previous report showed that two ERF members, *IbERF1* and *IbERF2*, are involved in different types of abiotic stress and in response to pathogens, and can activate the transcription of defence genes in tobacco [42]. *IbCBF3* can strengthen the drought and cold tolerance of the sweet potato [43]. Another *IbAP2/ERF* gene, *IbRAP2–12*, responded to salt and drought stress in transgenic Arabidopsis [22]. Recently, sweet potato *IbERF4* also played a vital role in regulating of abiotic stress [44].

In this study, through analysis of genome-wide biological information, the evolutionary characteristics of sweet potato *AP2/ERF* TFs were revealed. The expression profiles of *IbAP2/ERF* genes at different root developmental stages and under multiple forms of stress were further investigated by analysing RNA-seq and qRT-PCR data. This work lays a solid foundation for subsequent functional studies of the *AP2/ERF* gene family in the sweet potato.

## Methods

### Identification and classification of the *AP2/ERF* gene family in the sweet potato genome

Whole sweet potato genome data were downloaded from the Ipomoea Genome Hub (https://ipomoea-genome.org/). The AP2 domain (PF00847) was retrieved from the PFAM database (http://pfam.xfam.org/), and was used as the query for the HMM (hidden Markov model) search, which was conducted using the HMMER 3.0 programme with E < 1e^− 5^ as the threshold. Furthermore, the BLASTP programme with an e-value of 1e^− 5^ and identity of 50% as the threshold was used to search against the sweet potato protein dataset by using the AP2/ERF protein sequences of rice and Arabidopsis obtained from the plant transcription factor database (http://plntfdb.bio.uni-potsdam.de/v3.0/) as the query. Then, we used the NCBI-CDD web server (http://www.ncbi.nlm.nih.gov/Structure/cdd/wrpsb.cgi) and the SMART database (http://smart.embl-heidelberg.de/) to further verify the existence of the AP2 domain in all IbAP2/ERF proteins. The ExPASy server (http://www.expasy.org/) was used to calculate the MW (molecular weight) and PI (theoretical isoelectric point) of the retrieved proteins using the compute pI/Mw tool. The Cell-PLoc 2.0 web server (http://www.csbio.sjtu.edu.cn/bioinf/plant-multi/) was used to predict the subcellular localization of the retrieved proteins.

### Multiple sequence alignment and phylogenetic analysis

ClustalW with default parameters was used to perform the multiple sequence alignment of obtained AP2/ERF protein sequences. Phylogenetic and molecular evolutionary analyses were performed using MEGA7 with the neighbour-joining (NJ) algorithm. Phylogenetic trees were constructed using the retrieved conserved domains of AP2/ERF proteins. The bootstrap value was 1000. *IbAP2/ERF* genes were partitioned into three different groups based on the number of AP2 domains and the presence of B3 domains. The *ERF* subfamily was further subdivided into 12 groups (*DREB* A1-A6 and *ERF* B1-B6) based on the homologues of the corresponding genes in Arabidopsis.

### Sequence analysis

The Gene Structure Display Server (GSDS: http://gsds.gao-lab.org/index.php) was used to determine the exon-intron structure of these *IbAP2/ERF* genes. The structural differences among IbAP2/ERF proteins were investigated by studying the conserved protein domains. Additionally, the MEME programme was used to predict the conserved motifs of IbAP2/ERF proteins.

### Chromosome distribution, gene duplication and *cis*-acting elements in the promoters of *IbAP2/ERF* genes

From the genome annotation information, the chromosome distribution of all *IbAP2/ERF* genes was acquired and then confirmed by BLASTn search. Multiple collinear scanning toolkits (MCScanX) were used to evaluate gene replication events. Furthermore, we obtained the AP2/ERF protein sequences of *Arabidopsis thaliana*, *Oryza sativa*, *Manihot esculenta*, *Glycine max*, *Vitis vinifera* and *Zea mays* from the Phytozome database (https://phytozome.jgi.doe.gov/pz/portal.html#!search). Dual Synteny Plotter software (https://github.com/CJ-Chen/TBtools) was used to analyse the syntenic relationships among *AP2/ERF* genes in different selected plants. The 2000-bp genomic sequence was extracted from the upstream of the start codon of each *IbAP2/ERF* gene as the putative promoter region. Then, we used the PlantCARE database (http://bioinformatics.psb.ugent.be/webtools/plantcare/html) to predict *cis*-acting elements.

### Transcriptome data source and bioinformation analysis

High-throughput RNA-seq data (accession numbers PRJNA533954, PRJNA515432, PRJNA413661 and PRJNA631585) of the sweet potato were downloaded from the SRA database (http://www.ncbi.nlm.nih.gov/sra) and used to analyse the expression profiles of *IbAP2/ERF* genes by FPKM analysis. We used various quality parameters to assess the raw sequence data and used the NGS QC Toolkit (v2.3) [45] to filter the high-quality reads. Mapping onto the sweet potato genome of filtered high-quality reads was conducted by TopHat (v2.0.0) using the default parameters. The FPKM value and read counts of each sweet potato gene were obtained through Cufflinks (v2.0.2) using the mapped output. Read counts were used to detect differentially expressed genes with false discovery rate (FDR) < 0.01 and fold change > 2 through DESeq. The stage-specific/preferential genes in each stage were identified with the SS scoring algorithm, which compares the expression of a gene in a given stage with its maximum expression level in other stages as described previously [46]. A higher SS score of a gene in a particular stage signifies its more specific expression at that stage. A total of 15 RNA-seq datasets of sweet potato roots at different developmental stages, including fibrous roots (root diameters of approximately 1 mm) and storage roots (D1, D3, D5 and D10; root diameters of 1 cm, 3 cm, 5 cm and 10 cm, respectively), were used. Additionally, 9 RNA-seq datasets of sweet potato storage roots stored at 4 °C for 0 (control), 2, and 6 weeks were used to investigate the expression pattern of *IbAP2/ERF* genes under low temperature. Eight RNA-seq datasets of sweet potato leaves at 0 (control), 6, 12 and 24 h after 30% polyethylene glycol (PEG) treatment were used to explore the expression profile of *IbAP2/ERF* genes under drought stress. Moreover, 6 RNA-seq datasets of sweet potato roots at 0 (control) and 24 h after 150 mM NaCl treatment were used to analyse the transcript patterns of *IbAP2/ERF* genes in response to salt stress.

### Plant abiotic stress and low temperature treatment

Sweet potato (*Ipomoea batatas* L.) Cv. Taizhong6 seedlings were planted in early May in the Wushe Plantation for Transgenic Crops in Shanghai, China (31°13,948.0099 N, 121°28,912.0099E). Fibrous roots (S1, root diameter of 2 mm), pencil roots (S2, root diameter of 5 mm) and storage roots at two stages (S3 and S4; root diameters of 15 mm and 25 mm, respectively) were collected from the sweet potato plants in early November to cover the entire storage root initiation and development processes. Then, for low-temperature treatment, the collected storage roots (S4) were stored at 4 °C as described [47]. Tuberous roots were collected at 0 (control), 1, and 2 weeks after treatment. All samples were frozen in liquid nitrogen and stored at − 70 °C for mRNA extraction.

For abiotic treatments, sweet potato seedlings were incubated in quarter-strength Hoagland solution in a greenhouse (16 h/8 h of light/dark, 30 °C/22 °C day/night). The treatment assays were conducted as described in a previous report [25] with some modifications. Cold stress was performed by culturing seedlings at 4 °C, and the roots were collected. Dehydration and salt experiments were carried out by immersing the adventitious roots in 20% PEG6000 or 150 mM NaCl solutions, respectively, and the roots were harvested. All samples were collected at 0 (control), 1, 12, 24, and 48 h after each treatment and immediately frozen in liquid nitrogen for mRNA extraction.

### RNA extraction and quantitative real-time polymerase chain reaction (qRT-PCR) analysis

RNA Extraction Kits (TianGen, Beijing, China) were used to extract total RNA from samples according to the manufacturer’s instructions. Two micrograms of RNA was reverse-transcribed using ReverTra Ace qPCR RT Master Mix (TOYOBO, Shanghai, China). qRT-PCR analysis was performed as described earlier [48] with three biological replicates for each tissue sample and at least triplicates of each biological replicate. The gene-specific primers designed using Primer Express (v3.0) software are listed in Table S7. Each gene was normalized to the *β-Actin* internal control gene, and the fold change was calculated using the 2^−∆∆*CT*^ method.

### Analysis of subcellular localization

For the subcellular localization experiment, a construct coding for a g59127-GFP fusion protein was generated under the control of the CaMV 35S promoter, which was then introduced into tobacco leaves through *Agrobacterium*-mediated transformation. Finally, the leaves were observed under an Olympus FV1000 microscope (Olympus, Japan). The primers used in this study are listed in Table S7.

### Statistical analysis

Samples were collected from three independent plants. Data from at least three replicates are presented as the mean ± SD. Analysis of independent samples with Student’s *t*-test was performed using SPSS software, version 17 (SPSS Inc., Chicago, IL, USA). An alpha value of *P* < 0.05 was statistically significant.

## Results

### Identification of *AP2/ERF* family transcription factors in the sweet potato

All possible *IbAP2/ERF* genes were excavated from the sweet potato genome using a genome-wide search for AP2 domains including proteins. In total, 198 distinct *IbAP2/ERF* putative transcription factors were identified after removing redundant and alternative forms of the same gene (Table S1). The chromosome distribution results showed that these *IbAP2/ERF* genes were located on all 15 chromosomes in the sweet potato. In detail, *IbAP2/ERF* genes were most abundant on chromosome 7 with 23 *IbAP2/ERF* genes, while 22 and 17 *IbAP2/ERF* genes were distributed on the chromosomes 2 and 11, respectively. Otherwise, chromosome 14, with only 6 genes, had the fewest number of *IbAP2/ERF* TFs.

Gene characteristics were further investigated. Analysis of the coding sequence length (CDS) showed that *g60090* yielded the largest protein with 5064 bp (1687 amino acids), while *g532* yielded the smallest protein, with 309 bp (102 amino acids). The isoelectric point (pI) of these proteins ranged from 4.17 (g20630) to 11.65 (g54236), and the protein molecular weight (MW) ranged from 11.3 to 177.45 kDa. In addition, predicted subcellular localization analysis showed that the majority of *IbAP2/ERF* TFs were localized to the nucleus, with 157 genes, and 17 genes were localised to chloroplast. The remaining genes were predicted to be localised to mitochondria, peroxisomes, the plasma membrane and the cytoplasm.

### Phylogenetic relationship, gene structure and conserved motif analysis

**Fig. 1:**
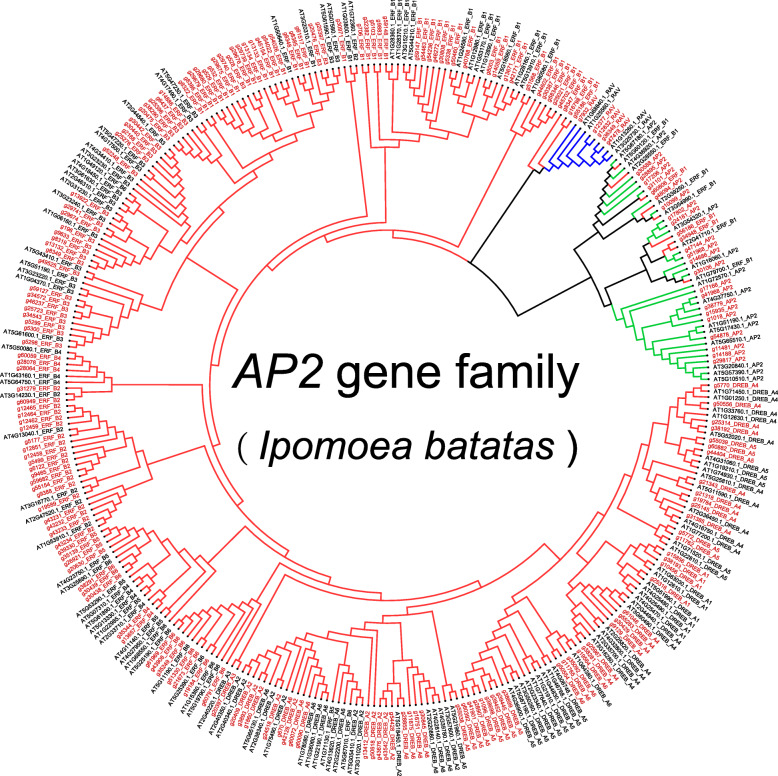
The phylogenetic tree constructed by the neighbor-joining method represents the relationships among AP2/ERF proteins of sweet potato and Arabidopsis. The proteins of sweet potato and Arabidopsis are marked in red and black respectively. Red, green and blue colored branches indicate *ERF*, *AP2* and *RAV* subfamily

To explore the phylogenetic relationship of sweet potato AP2/ERF proteins, the multiple sequence alignment results of 198 sweet potato AP2/ERF proteins and 141 Arabidopsis AP2/ERF proteins were used to construct a phylogenetic tree by using the neighbour-joining (NJ) method. All the *AP2/ERF* genes were clustered into three major clades (*ERF*, *AP2* and *RAV*) based on their domain composition (Fig. 1). There were 172 genes assigned to the *ERF* subfamily, which contains one single AP2 domain, and all these genes can be subdivided into two groups: DREB [A1 (4), A2 (10), A3 (1), A4 (15), A5 (14), A6 (11)] and ERF [B1 (38), B2 (20), B3 (40), B4 (5), B5 (4), B6 (10)], which is consistent with a previous report [25] (Table S2). The *AP2* subfamily had 21 genes with two AP2 DNA binding domains. Only 5 genes encoding a single AP2 domain and a B3 domain were grouped into the *RAV* family. Interestingly, the *g34948*, *g56186* and *g60806* genes belonged to the *ERF* subfamily members; however, these genes showed high similarity with the *AP2* subfamily.

**Fig. 2:**
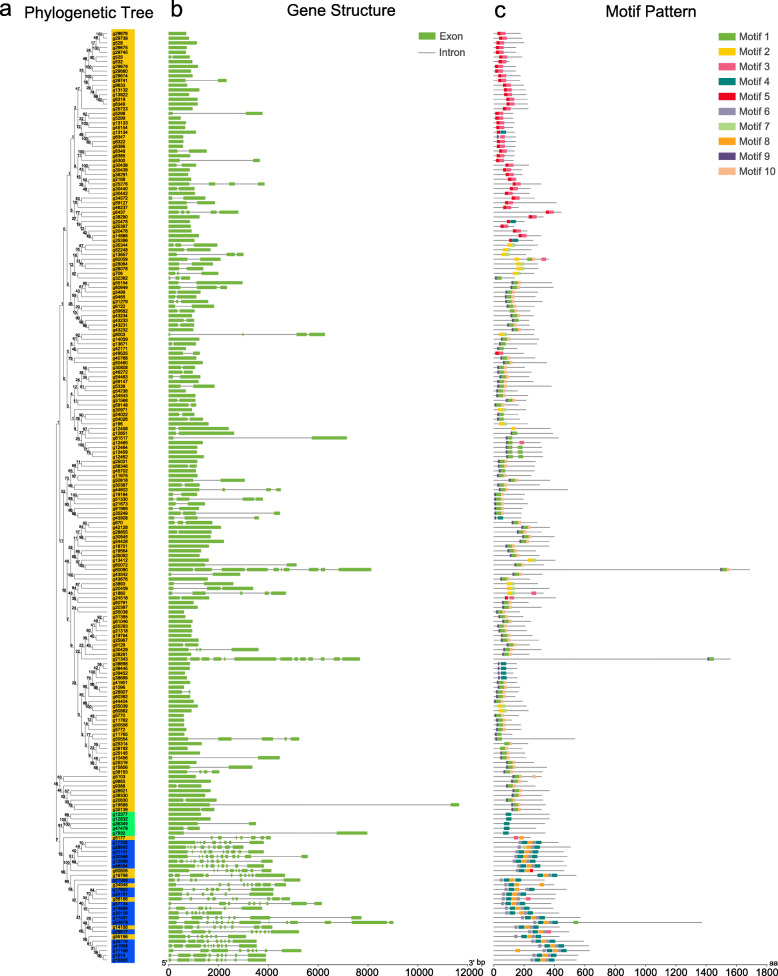
Architecture of phylogenetic tree, gene structure and protein conserved motifs in the *IbAP2/ERF* superfamily from sweet potato. (**a**) Phylogenetic relationships of IbAP2/ERF proteins in sweet potato. Members of *ERF*, *AP2* and *RAV* subfamily were filled in yellow, blue and green respectively. (**b**) Exon-intron structure of *IbAP2/ERF* genes. Green boxes indicate exons; black lines indicate introns. (**c**) The motif pattern of IbAP2/ERF proteins. The different colors represent different motifs with the number 1–10. The sequence information of each motif is provided in Additional file 1: Table S3. The protein length can be estimated using the scale at the bottom

To further understand the gene structural composition, the intron and exon structures of *IbAP2/ERF* genes were analysed by comparing the genomic DNA sequences (Fig. 2a, b). Most members of the *ERF* subfamily contained no or few introns, except for eight genes with intron numbers ranging from 7 to 12. Interestingly, 7 of those genes, including *g5177*, *g60806*, *g16798*, *g34948*, *g56186*, *g14188* and *g551886,* showed high similarity with the *AP2* subfamily. Additionally, the *RAV* subfamily contained no or one intron. Compared with the *ERF* and *RAV* subfamilies, the members of the *AP2* subfamily had at least 5 introns. Among *AP2* subfamily genes, *g54878* contained the most introns (14). The highly diverse gene structure indicated that there was extensive differentiation during the formation and evolution of the sweet potato genome.

Furthermore, characteristic region analysis of IbAP2/ERF proteins was conducted (Fig. S1). These proteins have a highly conserved AP2 domain, which is the typical pattern in the *AP2/ERF* family, especially in the *AP2* subfamily, which contains two conserved AP2 domains. In addition to the AP2 domain, *RAV* subfamily members also contained the B3 domain consisting of 100–120 amino acids. Moreover, other conserved regions were also detected in individual proteins. For example, the PRA1 domain, the ribosomal-S9 domain and the Metallophos domain were detected in the g5338 protein, g50554 protein and g54878 protein, respectively.

The conserved motifs of IbAP2/ERF proteins were further characterised by MEME software (Table S3). The results showed that a total of 10 conserved motifs were identified (Fig. 2a, c). Among these motifs, motifs 1, 2, 3, 4, 5, 6, 7 and 9 were located in the AP2 conserved domain regions. Besides, *ERF* subfamily members contained motifs 1, 2, 3, 4, 5, 6, 7, 8, 9 and 10, of which motif 9 was detected in most genes (100), and motif 6 was detected in the fewest genes (6). In the *AP2* subfamily members, motifs 1, 2, 3, 4, 6 and 7 were detected, of which motifs 2, 4 and 6 were found in almost all the members of the *AP2* subfamily. Motif 1 was detected in g54878, motif 3 in g29817, and motif 7 in g28895. In the *RAV* subfamily, motif 4 was the only shared motif. Generally, many conserved motifs detected in these IbAP2/ERF proteins may participate in the expression regulation of genes with the potential DNA binding sites, which can be further examined. The similar composition of gene structure and conserved motifs in a specific subfamily further verified the reliability of the phylogenetic tree and clustering.

### Chromosome distribution, gene duplication and synteny analysis of the *IbAP2/ERF* gene family

**Fig. 3:**
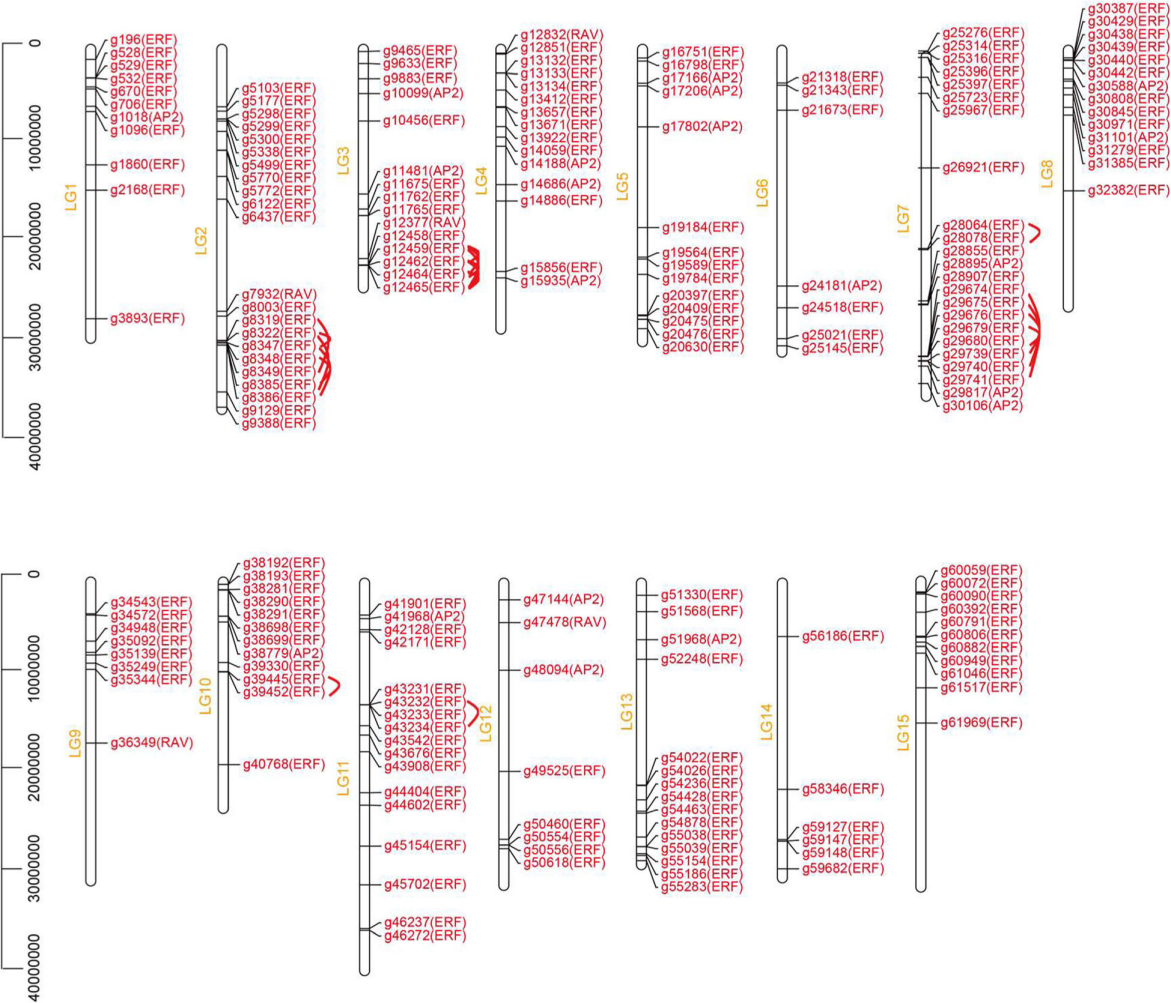
Chromosomal distribution of sweet potato *IbAP2/ERF* genes. The chromosome number is marked on the left of each chromosome. The red lines represent duplicated *IbAP2/ERF* gene pairs

To investigate the chromosome distribution of the *IbAP2/ERF* genes, the latest sweet potato genome database was used for analysis. A total of 198 *IbAP2/ERF* genes were distributed unevenly on 15 sweet potato chromosomes (Fig. 3). Chromosome 7 had the largest number of *IbAP2/ERF* TFs (27 genes), which accounted for approximately 11.6% of the total number of *IbAP2/ERF* genes. Chromosome 2 contained 22 *IbAP2/ERF* genes, which accounted for approximately 11% of the total number of *IbAP2/ERF* genes, while the smallest number of *IbAP2/ERF* TFs was found on chromosome 14, with 6 genes. The *ERF* subfamily was detected on all chromosomes, of which chromosomes 14 and 15 contained only *ERF* subfamily members. In addition, no *AP2* subfamily members were found on chromosomes 2, 9, 14 or 15, while the members of the *RAV* subfamily were distributed on chromosomes 2, 3, 4, 9 and 12. Furthermore, some *IbAP2/ERF* TFs that have similar conserved structures were localised on the same chromosome, which has also been observed in *Arabidopsis thaliana* [5], *Vitis vinifera* [49], Chinese cabbage [50] and Tartary buckwheat genomes [51], indicating that ancestral polyploidy events may result in these homologous fragments. In addition, we analysed the duplication events of *IbAP2/ERF* genes because gene duplication is a key mechanism in gene expansion and the emergence of novel functions. Tandem replication was defined as 200 kb-range chromosomal regions that included more than one homologous gene. Six *IbAP2/ERF* gene clusters containing twenty-six tandem duplicated genes were identified in sweet potato linkage groups (LGs) 2, 3, 7, 10 and 11. LG7 had two clusters, one of which contained the most genes (8 genes), indicating hot spots of *IbAP2/ERF* gene distribution. Interestingly, each cluster contained only genes belonging to the *ERF* subfamily.

**Fig. 4:**
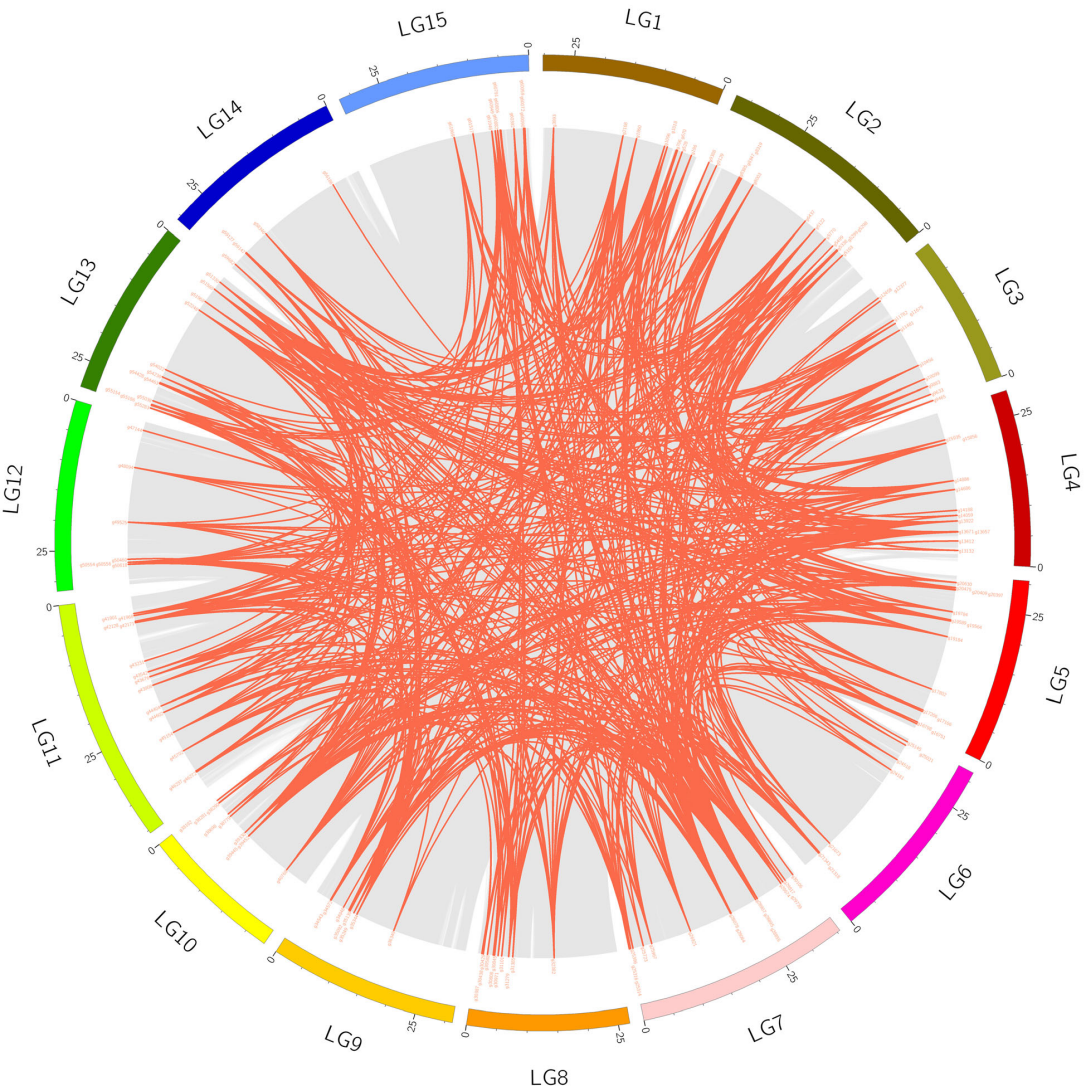
Interchromosomal relationships of sweet potato *IbAP2/ERF* genes. Grey lines indicate all syntenic blocks in the sweet potato genome. Red lines indicate collinear blocks of *IbAP2/ERF* genes in the sweet potato genome

Apart from tandem duplication events, we also found many pairs of segmental duplications in the sweet potato chromosomes (Fig. 4), since the analysis of homologous genes is significant in exploring the kinship of evolution. Several pairs of homologous genes were found on different sweet potato chromosomes, further confirming that the *IbAP2/ERF* gene family is highly conserved. According to the above data, some *IbAP2/ERF* genes might be the result of gene replication, which might be the main evolutionary driving force of *IbAP2/ERF* genes.

### Evolutionary analysis of *AP2/ERF* genes in the sweet potato and several different species

**Fig. 5:**
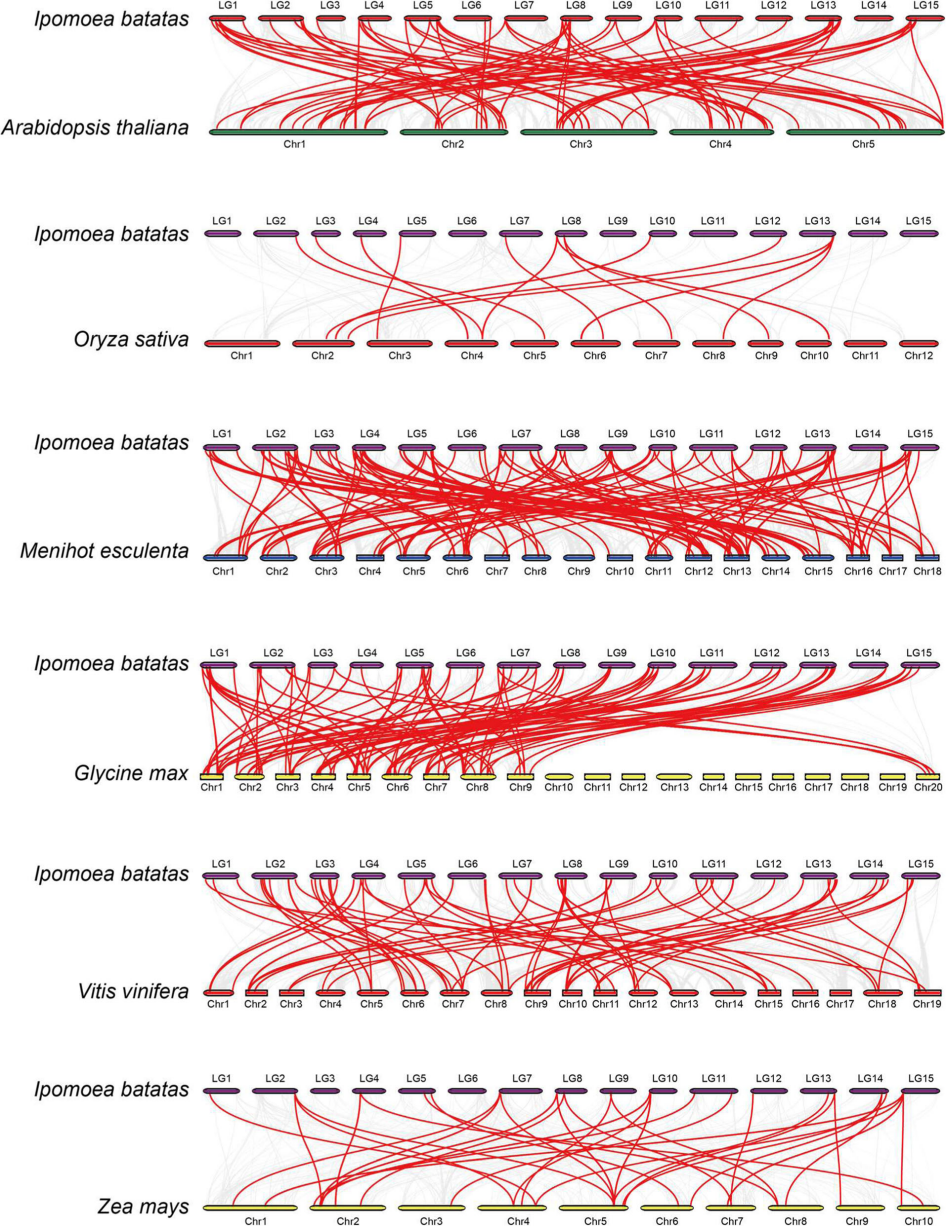
Syntenic analysis of *AP2/ERF* genes between sweet potato and six representative plant species. Gray lines in the background indicate the collinear blocks within sweet potato and other plant genomes, whereas red lines highlight syntenic *AP2/ERF* gene pairs

Since the phylogenetic mechanisms of the *IbAP2/ERF* family were uncertain, we constructed syntenic maps of the sweet potato compared with six different species including three monocotyledonous plants (grape, corn, rice) and three dicotyledonous plants (soybean, Arabidopsis, and cassava). The results showed that the *AP2/ERF* genes in the sweet potato have homologous genes in these reference plants, of which *Manihot esculenta* had the most syntenic conservation (158 syntenic gene pairs located on chr1 - chr18), followed by *Arabidopsis thaliana* (119 orthologous gene pairs distributed on chr1 - chr5) and *Glycine Max* (109 syntenic gene pairs distributed on chr1 – chr9 and chr20) (Fig. 5). When comparing between the sweet potato and *Manihot esculenta,* the syntenic results of *AP2/ERF* genes showed that *g13132*, *g13922*, *g46272*, *g55039* and *g60882* were connected with more than two orthologous gene pairs, indicating that these genes might be of great significance in *AP2/ERF* family evolution (Table S4). According to the above results, sweet potato *AP2/ERF* genes are closer to those in cassava and may evolve from a common ancestor in various plants.

### *Cis*-acting elements of sweet potato *IbAP2/ERF* genes

**Fig. 6:**
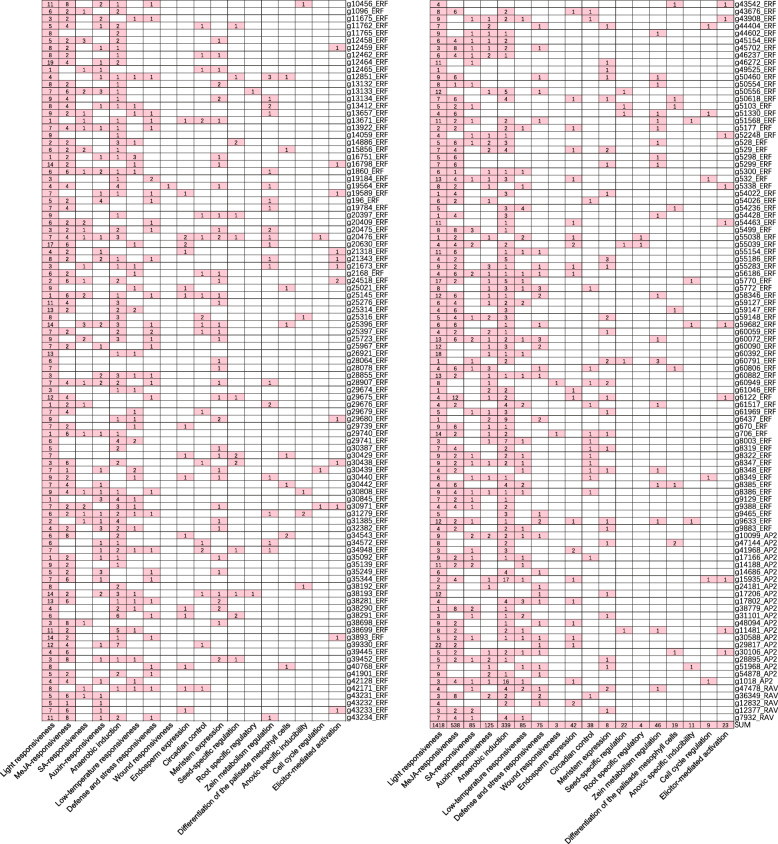
Predicted *cis*-acting elements in the promoter of the *IbA2/ERF* genes. The 2.0 kb region upstream of ATG was analysed using the PlantCARE software

To further infer the potential function of sweet potato *IbAP2/ERF* genes, *cis*-acting elements were analysed using the promoters of these genes (Fig. 6). Many *cis*-acting elements, such as hormone-responsive, stress-responsive and light-responsive elements, were observed in the promoters of *IbAP2/ERF* genes. Light-responsive elements (1418) were the most enriched *cis*-elements in the promoters of *IbAP2/ERF* genes. Hormone-responsive elements (748), such as methyl jasmonate (MeJA, 538), SA (85) and auxin (125), were often detected in the promoters of *IbAP2/ERF* genes. The promoters also included stress-related elements for anaerobic induction (339), low-temperature responsiveness (85), defence and stress responsiveness (75) and wound responsiveness (3). Additionally, endosperm expression (42), circadian control (38) and cell cycle regulation (9) promoter elements were also detected. These results implied that the *IbAP2/ERF* genes may be regulated through various *cis*-acting elements and play significant roles during plant development and stress responses.

### Expression patterns of *IbAP2/ERF* genes during root development and under multiple forms of abiotic stress

**Fig. 7:**
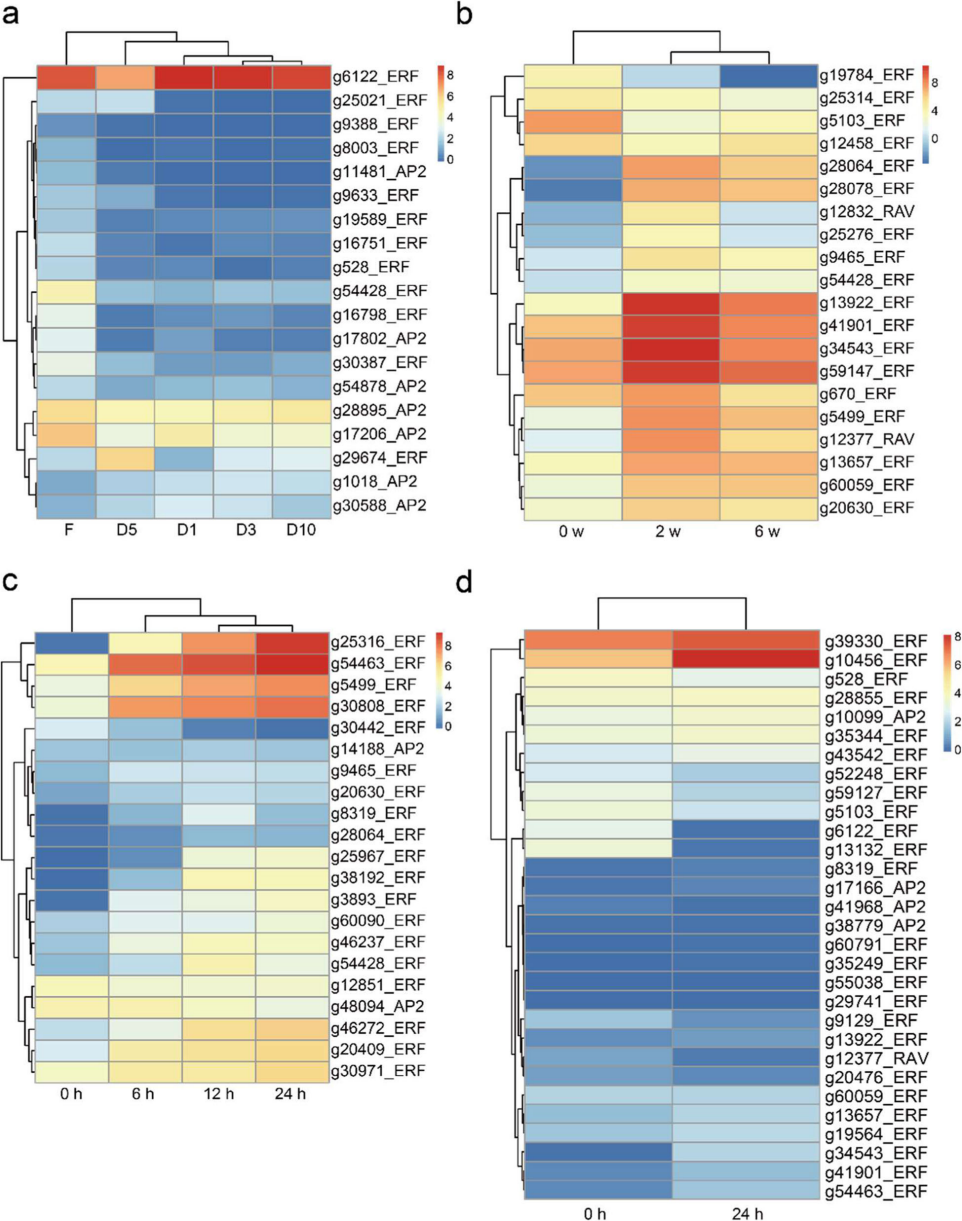
The expression profiles of *IbAP2/ERF* genes in the sweet potato at different root developmental stages and under multiple forms of stress analysed by the available RNA-Seq data. (**a**) The expression profiles of *IbAP2/ERF* genes in the sweet potato roots at different developmental stages. F, fibrous roots; D1, the pencil roots (diameter: 1 cm); D3, the storage roots (diameter: 3 cm); D5, the storage roots (diameter: 5 cm); D10, the storage roots (diameter: 10 cm). (**b**) The expression profiles of *IbAP2/ERF* genes in the root under cold stress. w, week. (**c**) The expression profiles of *IbAP2/ERF* genes in the leaves under 30% PEG treatment. h, hour. (**d**) The expression profiles of *IbAP2/ERF* genes in the roots under salt treatment. h, hour. For each row, blue and red correspond to low and high values of gene expression, respectively

To determine the functional roles of *IbAP2/ERF* genes during root development, the expression profiles of these genes at different root developmental stages (F, fibrous roots; D1, pencil roots with a diameter of 1 cm; D3, storage roots with a diameter of 3 cm; D5, storage roots with a diameter of 5 cm; D10, storage roots with a diameter of 10 cm) were analysed using RNA-seq data through FPKM analysis. In the results, 191 *IbAP2/ERF* genes were examined in these data, and the expression levels of these genes had high variance, indicating that the *IbAP2/ERF* genes had multiple potential functions in sweet potato root development (Fig. 7a and Table S5). Generally, among these genes, 44, 7, 6 and 2 genes were relatively highly expressed in the F, D1, D5 and D10 stages, respectively. In detail, *g15856*, *g25314*, *g38193*, *g40768*, *g14059*, *g20630* and *g25967* were specifically expressed in the early developmental stages (D1), with extremely low expression levels in the other stages. We presumed that these genes may mainly affect the early development of roots and may be used as marker genes during early root developmental stages. In addition, several genes showed relatively high expression at each stage of root development, such as *g31279*, *g6122*, *g54463*, *g59147*, *g60949*, *g60090*, *g34543*, *g30808*, *g670* and *g20475*, indicating that they may play indispensable roles in regulating tuber development. However, *g38291*, *g39445*, *g39452*, *g38698*, *g47478*, *g51568*, *g6437* and *g20397* were not expressed in any of the tested samples.

We found low-temperature responsive elements in the promoters of *IbAP2/ERF* genes, indicating that *IbAP2/ERF* TFs might play indispensable roles in responding to low temperatures. To further analyse the physiological function of *IbAP2/ERF* genes under cold stress, the available RNA-seq data of sweet potato roots under cold stress were used to study the expression profiles of these genes (Fig. 7b and Table S5). In the results, 56 *IbAP2/ERF* genes were detected, and there were 29 differentially expressed genes, of which 16 genes were upregulated and 4 genes were downregulated under cold stress at 2 weeks. The expression level of *g60059* increased quickly under cold stress at 2 weeks and increased continuously to 6 weeks. A large proportion of TFs, such as *g13922*, *g41901*, *g34543*, *g59147*, *g670*, *g5499* and *g12377*, also increased quickly under cold stress at 2 weeks, but their expression levels irregularly changed at 6 weeks. The expression levels of *g19784* and *g25314* decreased quickly under cold stress at 2 weeks and decreased continuously to 6 weeks. Additionally, some genes, such as *g5103* and *g12458* exhibited decreased expression patterns under cold stress at 2 weeks, but their expression levels increased at 6 weeks. These results implied that the *IbAP2/ERF* TFs might play indispensable roles in the response to cold stress.

For dehydration stress, the expression patterns of *IbAP2/ERF* genes were investigated by RNA-seq data of sweet potato leaves under 30% PEG treatment (Fig. 7c and Table S5). A total of 52 *IbAP2/ERF* genes were detected, wherein 18 genes including 17 upregulated genes and 1 downregulated gene were differentially expressed at 6 h. There was only one gene (*g54428*) differentially expressed at 12 h. At 24 h, 5 genes and 2 genes were upregulated or downregulated, respectively. Among these genes, *g54428* was differentially expressed at all the time points, implying that it might contribute to the sweet potato response to drought stress. Additionally, 155 *IbAP2/ERF* genes were examined in the RNA-seq data of sweet potato roots under salt stress (Fig. 7d and Table S5). The transcript level of 21 genes were increased at 24 h, while there were 9 downregulated genes at 24 h. *g35344* and *g43542* might be key regulators of the response to salt stress because they exhibited the highest induction level under salt stress with approximately a 64-fold change.

**Fig. 8:**
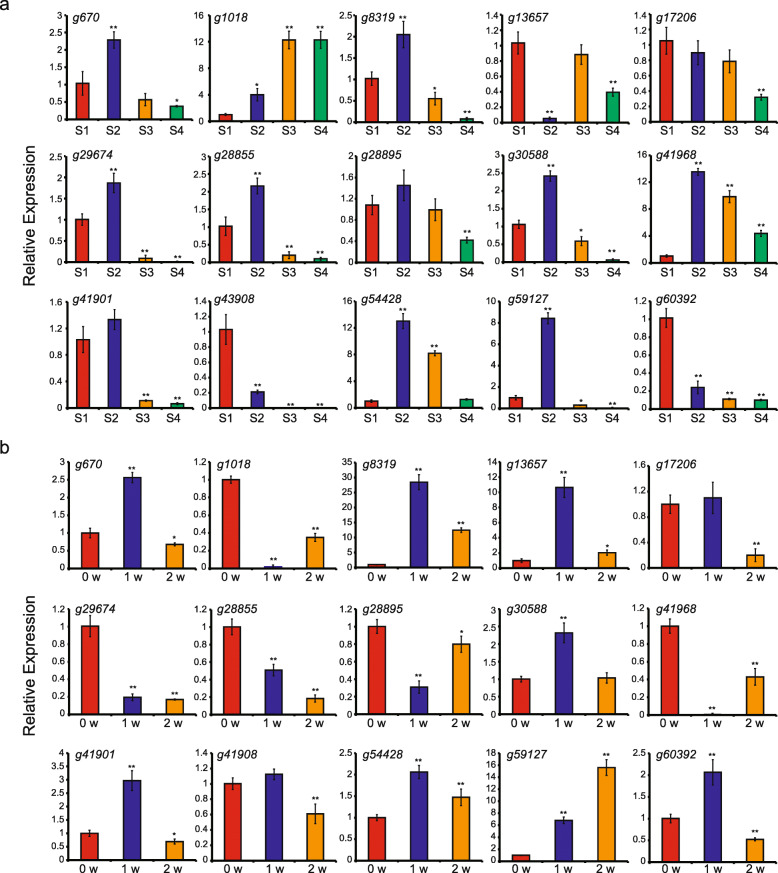
The expression profiles of *IbAP2/ERF* genes in the roots at different developmental stages (a) and during storage process at low temperature (b) analyzed by qRT-PCR. Fibrous roots (S1, root diameter of 2 mm), pencil roots (S2, root diameter of 5 mm) and storage roots at two stages (S3 and S4; root diameters of 15 mm and 25 mm respectively) from the sweet potato plants were harvested at six months after planting. Then, the collected storage roots were stored at 4 °C. Tuberous roots were collected at 0 (control), 2, and 6 weeks after low temperature treatment. The expression data were normalized to 1 in S1 and unstressed plants (0 w). Bars represent the mean of replicates ± standard error. * and ** indicate a significant difference at *P* < 0.05 and < 0.01, respectively, determined by Student’s *t*-test

**Fig. 9:**
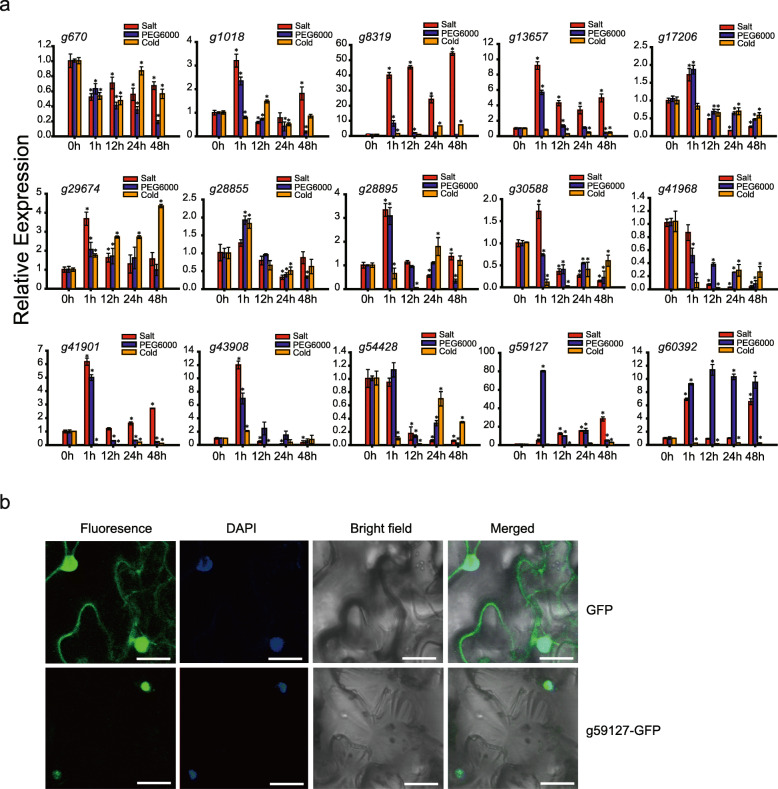
The expression profiles of *IbAP2/ERF* genes detected by qRT-PCR under various types of abiotic stress and the subcellular localization of g59127 protein. (**a**) The expression profiles of *IbAP2/ERF* genes detected by qRT-PCR under NaCl, dehydration, and cold stress. The sweet potato seedlings were submerged in 150 mM NaCl and 20% (w/v) PEG6000 solutions, respectively, and then adventitious roots were harvested. Cold assays were carried out by incubating the seedlings at 4 °C, and then roots were collected. The expression data were normalized to 1 in unstressed plants (0 h). Bars represent the mean of replicates ± standard error. * indicates a significant difference at *P* < 0.05, determined by Student’s *t*-test. (**b**) Analysis of the subcellular localization of g59127 protein. Localization of g59127-YFP fusion protein in tobacco (*Nicotiana* L.) cells. *g59127*-YFP expression was visualized using a confocal laser scanning microscope. Bar, 20 μm

To validate the RNA-seq results, we performed qRT-PCR analysis of 15 main abiotic stress-induced genes with high transcriptional expression levels based on the RNA-seq data (Fig. 8a-b and Fig. 9a). The results showed that abiotic stress can lead to dramatic alterations in these selected genes. The expression profiles revealed by qRT-PCR were similar to those obtained by RNA-seq (Fig. 7a-d), indicating the accuracy of RNA-seq data and the potential contribution of the tested genes to root development and in response to abiotic stress. Among these genes, *g670*, *g8319*, *g17206*, *g29674*, *g28855*, *g28895*, *g30588*, *g41901*, *g43908*, *g59127* and *g60392* were more highly expressed at early root developmental stages (S1 and S2), while *g1018* was more highly expressed at later stages (S3 and S4), indicating that different genes might play various roles during the root developmental process (Fig. 8a). Under salt stress, *g8319* was the most significantly induced (approximately 55-fold), followed by *g59127* (30-fold), *g13657* (9-fold), *g41901* (6-fold) and *g29674* (3.5-fold). However, *g670*, *g41968* and *g54428* expression was inhibited, implying that these genes might be key regulators of the response to salt stress (Fig. 9a). For dehydration stress, the highest induction level was observed in *g59127* at 80-fold. Expression of *g60392* also increased (approximately 12-fold), whereas that of *g670*, *g30588* and *g41968* was inhibited (Fig. 9a). For cold stress, *g8319* had the most significant induction level (at approximately 6-fold), followed by *g29674* (at approximately 4-fold). In contrast, many of the analysed genes, including *g670*, *g13657*, *g17206*, *g30588*, *g41968*, *g41901*, *g54428* and *g60392*, were inhibited when subjected to cold stress (Fig. 9a). Collectively, the significant and diverse expression patterns of these genes implied that they might play a role in responding to abiotic stress.

### Analysis of subcellular localization of g59127 protein

Because the transcriptional expression of the *g59127* gene showed obvious alterations during root development and could be markedly affected by most forms of abiotic stress, including cold, dehydration and salt stress, it was selected for further molecular characterisation analyses. The g59127 protein was predicted to be in the nucleus (Table S1), and a vector with the translational fusion of g59127 to GFP was constructed to confirm this result. As shown in Fig. 9b, the free GFP protein was both nuclear and cytoplasmic, but the g59127-GFP fusion protein was only displayed in the nucleus, which was consistent with the bioinformatics results.

## Discussion

The *AP2/ERF* superfamily is one of the largest families of plant-specific transcription factors and plays important roles in a variety of biological processes. Many works have been performed to identify members of the *AP2/ERF* superfamily in several plants with sequenced genomes, such as Arabidopsis [5], maize [52], peach [53] and foxtail millet [54]. Nevertheless, no detailed study of this superfamily has been carried out in the sweet potato at the whole genome level until now. In this study, extensive identification of *AP2/ERF* genes throughout the sweet potato genome was conducted. A total of 198 sweet potato *AP2/ERF* genes were discovered (Table S1), accounting for 0.31% of all the sweet potato genes, which is lower than the results observed in rice (0.43%), maize (0.44%), foxtail millet (0.44%) and *Brachypodium distachyon* (0.45%). Compared with other plants, the *AP2/ERF* gene number in the sweet potato (198) was greater than that in barley (121), longan (125), tomato (146), Arabidopsis (148) and rice (167) but lower than that in poplar (202) and Chinese cabbage (291). It has been reported that the number of *AP2/ERF* genes is determined by the number of ERF subfamily members to a certain extent [55]. There were 172 *ERF* subfamily genes in the sweet potato and 122, 132 and 158 in Arabidopsis, rice and maize, respectively. Gene evolution and duplication have been revealed to cause this variance in plants [56, 57]. Additionally, there was no significant variance in the number of *AP2* and *RAV* family members among these plant species, with values of 24, 25, 26, 28 and 34 in Arabidopsis, maize, sweet potato, *Brachypodium distachyon* and rice, respectively.

In the gene intron/exon structure of the 198 *IbAP2/ERF* genes, the *AP2* subfamily had more introns, while the *ERF* subfamily had fewer introns, and no intron was found in the *RAV* subfamily (Fig. 2a, b), which resembles that of *AP2/ERF* genes in other plant species, including cauliflower and radish [58, 59]. Some studies have revealed that plant evolution is related to intron number and distribution [6], and the intron number of *ERF* subfamily genes is probably lost during plant evolution [60, 61]. Herein, no intron was observed in 92 of the 172 *ERF* subfamily members (53%), but a higher number was reported for Tartary buckwheat [51]. The variance of the gene structure among *AP2*, *ERF* and *RAV* subfamily members indicated that there might have been extensive differentiation and numerous functional discrepancies between these subfamilies during the evolution of the sweet potato genome. In addition, conserved domains and motifs play important roles in regulatory functions, which are associated with transcriptional activity, DNA binding and protein interactions [62, 63]. Previous reports have shown that in addition to an N-terminal DNA-binding domain, the C-terminal activation domain of AP2/ERF proteins can regulate the transcription of their target genes in Arabidopsis and rice [3]. AP2/ERF genes with ERF-associated amphiphilic repression (EAR) motifs (LxLxL or DLNxxP sequence) or B3 repression domains (BRD, R/KLFGV sequence) have a repressive effect on their target genes [64, 65]. The EDLL motif identified from *AtERF98* can override the repressive effect mediated by the EAR motif [66]. In this study, 41 *IbAP2/ERFs* had the EAR motif, and 4 *IbAP2/ERFs* had the B3 motif (Table S6), implying that these genes might be involved in negative regulatory functions. Additionally, the EDLL motif was detected in 4 *IbAP2/ERFs* (Table S6), suggesting that the regulation of these genes may be complex, but further experimental verification is needed. Moreover, another 10 motifs were found in IbAP2/ERF proteins based on the MEME results: eight of the 10 motifs (motifs 1–7, 9) were related to the AP2 domain, and only 2 conserved motifs were located outside the AP2 domain (Fig. 2, Fig. S1 and Table S3). The *ERF* subfamily members had all 10 motifs, of which motif-9 was shared by most genes (100). Motif-9 was detected in the AP2 domain and enriched many DNA binding sites, indicating that this motif may be essential for the DNA binding abilities of these TFs [67]. Additionally, *AP2* subfamily members harboured numerous motifs, ranging from 1 to 4 and 6 to 7, whereas the *RAV* family members only had motif-4. Based on these results, although high conservation was observed in some motifs of the *IbAP2/ERF* family, the unique motifs of different subgroups might be involved in more special functions in each *IbAP2/ERF* subfamily, and their functions require more work to clarify.

The latest sweet potato genome database was used to analyse the chromosome distribution of the *IbAP2/ERF* genes, and these genes were unevenly anchored on 15 chromosomes (LGs) (Fig. 3 and Fig. 4). Hot regions existed in most chromosomes, which indicated that *IbAP2/ERF* gene family expansion might be caused by tandem duplication and segmental duplications, which is in accordance with previous studies [55, 68]. In total, 26 paralogous pairs were found in the sweet potato, more were discovered in rice (41), Arabidopsis (51) and grape (76), and less were discovered in jujube (18). Furthermore, using MCScanX, there were 38,290 collinear gene pairs in the sweet potato genome, and 683 *IbAP2/ERF* collinear gene pairs were recognised, indicating that the sweet potato genome experienced a whole genome duplication event that might also underlie the expansion of the *IbAP2/ERF* family (Fig. 5 and Table S4).

Previous reports have shown that *AP2/ERF* TFs can be potential candidates for crop improvement because they are key regulators in different plant development processes and various stress responses [43, 69–72]. Nevertheless, *IbAP2/ERF* gene functions in the sweet potato are still not well known, and it is essential to analyse the transcriptional regulation of *IbAP2/ERFs* to utilise them to improve the quality and abiotic stress tolerance of the sweet potato. Here, we systematically analysed the expression profiles of these genes during root development and under multiple types of stress to determine their potential functions in biological processes. In this study, a total of 191 *IbAP2/ERFs* were expressed at different root developmental stages, implying that they might be widely associated with the regulation of root growth and development (Fig. 7a and Table S5). Furthermore, prominent temporal expression patterns of *IbAP2/ERF* genes were also observed. Forty-four *IbAP2/ERF* genes were specifically expressed in fibrous roots and 2 *IbAP2/ERF* genes showed preferential expression in the mature storage root (D10). Additionally, the expression levels of 60 *IbAP2/ERF* genes increased gradually during root development, indicating that these genes might play crucial roles in the process. In particular, *g20630* showed a continuous upregulation profile, and its homologous gene *AtCRF3* was reported to regulate lateral root development in Arabidopsis [73], implying that *g20630* might have a similar function in sweet potato root development, thus confirming the reliability of our results. Furthermore, *AP2/ERF* TFs were reported to regulate the expression of target genes that respond to stress by binding to GCC-box or DRE motifs [74, 75]. Our results showed that there were 85 low-temperature responsive and 75 defence- and stress-responsive *cis*-elements in the promoter regions of *IbAP2/ERF* genes (Fig. 6). Moreover, compared to the control, *IbAP2/ERF* genes were specifically induced or repressed under multiple types of stress (Fig. 7b-d, Fig. 8b, Fig. 9a and Table S5). In particular, the expression of *g25316*, which encodes the IbDREB1/IbCBF3 protein, showed a remarkable reduction under cold stress and a significant increase under drought stress, which is consistent with a previous study [41, 43]; and the function of its homologue *AtCBF3* in Arabidopsis [76], further confirming the reliability of our results. *IbERF1* (*g35249*) and *IbERF7* (*g55038*) was induced by salt stress, which is in accordance with a previous report [25, 42]. Moreover, *IbRAP2–12* (*g60949*) showed increased patterns under both drought and salt stress, which is similar to published results [22]. *IbERF4* (*g30808*) showed a similarly increased profile under drought stress compared with a previous report [44]. In addition, a previous report showed that *AtERF113* (*RAP2.6 L*) can be activated by drought and salt stress, and enhance the tolerance to these stressors in Arabidopsis [77]. Our results found that the homologous genes of *AtERF113* in the sweet potato, including *g28064*, *g60059* and *g52248*, showed similar expression patterns to those in a previous report [25], indicating that these genes may play similar roles in the sweet potato stress response. Based on the above data, we speculated that *cis*-elements might be crucial regulatory factors for the spatial and temporal expression of *IbAP2/ERF* genes, which could form a complex regulatory network with other functional proteins during development and stress response processes [78]. These identified developmental stage-specific and stress-induced *IbAP2/ERF* genes might be valuable candidates for systematic functional investigations of these genes in the sweet potato and other tuberous crops.

The bioinformatics analysis of the subcellular localization of *IbAP2/ERF* TFs showed that most of these genes were in the nucleus (157), while others were distributed in chloroplasts (17), the cytoplasm (15), mitochondria (6) and peroxisomes (3) (Table S1). The results of the subcellular localization experiment verified that g59127 localised to the nucleus, which was in line with predicted results. In summary, our present study identified and characterised *IbAP2/ERF* TFs in the sweet potato. By conducting a genome-wide search, 198 *IbAP2/ERF* TFs were identified. The phylogenetic relationship, exon-intron structure, conserved motif composition, chromosome distribution and gene duplication of these *IbAP2/ERF* TFs were systematically discussed and compared. *IbAP2/ERFs* could be clustered into three major subfamilies, which was consistent with the number of AP2 domains and gene structure. The *cis*-acting elements in the promoter regions of the *IbAP2/ERF* genes were analysed, and we further clarified the expression patterns of these genes at different root developmental stages and under multiple forms of abiotic stress. Several storage root developmental stage-specific or abiotic stress-responsive *IbAP2/ERF* TFs were identified, which might be ideal candidate genes for further functional study of the corresponding biological processes and the development of high-quality and stress-tolerant sweet potatos by genetic engineering. Our study originally discovered the components, structures, evolution and expression profiles of the *IbAP2/ERF* superfamily, which could facilitate further functional analyses of *IbAP2/ERF* genes and a better understanding of the molecular mechanisms in developmental processes and stress responses in the sweet potato.

## Supplementary Information

**Additional file 1: Table S1.** Complete list of *IbAP2/ERF* genes identified in the sweet potato genome. **Table S2.** One-to-one corresponding relationships of *IbAP2/ERF* genes between this study and previous report. **Table S3.** Analysis and distribution of conserved motifs in sweet potato IbAP2/ERF proteins. **Table S4.** One-to-one orthologous relationships between sweet potato and other species. **Table S5.** Expression profiles of *IbAP2/ERF* genes at different root developmental stages and under multiple types of abiotic stress. **Table S6.** Analysis and distribution of known motifs in sweet potato IbAP2ERF proteins. **Table S7.** Primer pairs used in this study.

**Additional file 2: Fig. S1.** Phylogenetic relationships and conserved domains in IbAP2/ERF proteins from sweet potato.

## Data Availability

Public datasets from SRA database (http://www.ncbi.nlm.nih.gov/sra) were used in this study. For RNA-seq data, we used root development data of sweet potato (PRJNA515432), chilling response data (PRJNA533954), drought response data (PRJNA413661) and salt response data (PRJNA350623). All of the datasets supporting the results of this article are included within the article and its additional files. The collection of sweet potato materials was permitted and complied with relevant institutional, national, and international guidelines and legislation.

## References

[1] Jofuku KD, Omidyar PK, Gee Z, Okamuro JK (2005). Control of seed mass and seed yield by the floral homeotic gene APETALA2. Proc Natl Acad Sci U S A.

[2] Wessler SR (2005). Homing into the origin of the AP2 DNA binding domain. Trends Plant Sci.

[3] Nakano T, Suzuki K, Fujimura T, Shinshi H (2006). Genome-wide analysis of the ERF gene family in Arabidopsis and rice. Plant Physiol.

[4] Cao ZF, Li J, Chen F, Li YQ, Zhou HM, Liu Q (2001). Effect of two conserved amino acid residues on DREB1A function. Biochemistry (Mosc).

[5] Sakuma Y, Liu Q, Dubouzet JG, Abe H, Shinozaki K, Yamaguchi-Shinozaki K (2002). DNA-binding specificity of the ERF/AP2 domain of Arabidopsis DREBs, transcription factors involved in dehydration- and cold-inducible gene expression. Biochem Biophys Res Commun.

[6] Hu L, Liu S (2011). Genome-wide identification and phylogenetic analysis of the ERF gene family in cucumbers. Genet Mol Biol.

[7] Wu L, Zhang Z, Zhang H, Wang XC, Huang R (2008). Transcriptional modulation of ethylene response factor protein JERF3 in the oxidative stress response enhances tolerance of tobacco seedlings to salt, drought, and freezing. Plant Physiol.

[8] Licausi F, Ohme-Takagi M, Perata P (2013). APETALA2/ethylene responsive factor (AP2/ERF) transcription factors: mediators of stress responses and developmental programs. New Phytol.

[9] Nole-Wilson S, Krizek BA (2000). DNA binding properties of the Arabidopsis floral development protein AINTEGUMENTA. Nucleic Acids Res.

[10] Gong W, He K, Covington M, Dinesh-Kumar SP, Snyder M, Harmer SL, Zhu YX, Deng XW (2008). The development of protein microarrays and their applications in DNA-protein and protein-protein interaction analyses of Arabidopsis transcription factors. Mol Plant.

[11] Sohn KH, Lee SC, Jung HW, Hong JK, Hwang BK (2006). Expression and functional roles of the pepper pathogen-induced transcription factor RAV1 in bacterial disease resistance, and drought and salt stress tolerance. Plant Mol Biol.

[12] Jofuku KD, den Boer BG, Van Montagu M, Okamuro JK (1994). Control of Arabidopsis flower and seed development by the homeotic gene APETALA2. Plant Cell.

[13] Krizek B (2009). AINTEGUMENTA and AINTEGUMENTA-LIKE6 act redundantly to regulate Arabidopsis floral growth and patterning. Plant Physiol.

[14] Xu ZS, Chen M, Li LC, Ma YZ (2011). Functions and application of the AP2/ERF transcription factor family in crop improvement. J Integr Plant Biol.

[15] Jaglo-Ottosen KR, Gilmour SJ, Zarka DG, Schabenberger O, Thomashow MF (1998). Arabidopsis CBF1 overexpression induces COR genes and enhances freezing tolerance. Science.

[16] Wang M, Dai W, Du J, Ming R, Dahro B, Liu JH (2019). ERF109 of trifoliate orange (Poncirus trifoliata (L.) Raf.) contributes to cold tolerance by directly regulating expression of Prx1 involved in antioxidative process. Plant Biotechnol J.

[17] Qin F, Kakimoto M, Sakuma Y, Maruyama K, Osakabe Y, Tran LS, Shinozaki K, Yamaguchi-Shinozaki K (2007). Regulation and functional analysis of ZmDREB2A in response to drought and heat stresses in Zea mays L. Plant J.

[18] Hong B, Ma C, Yang Y, Wang T, Yamaguchi-Shinozaki K, Gao J (2009). Over-expression of AtDREB1A in chrysanthemum enhances tolerance to heat stress. Plant Mol Biol.

[19] Wang XM, Chen XF, Liu Y, Gao HW, Wang Z, Sun GZ (2011). CkDREB gene in Caragana korshinskii is involved in the regulation of stress response to multiple abiotic stresses as an AP2/EREBP transcription factor. Mol Biol Rep.

[20] Ito Y, Katsura K, Maruyama K, Taji T, Kobayashi M, Seki M, Shinozaki K, Yamaguchi-Shinozaki K (2006). Functional analysis of rice DREB1/CBF-type transcription factors involved in cold-responsive gene expression in transgenic rice. Plant Cell Physiol.

[21] Liang CL, Li YN, Zhang XP, Song Y, Wang W, Fang J, Cui WM, Jia XD (2012). Immunotoxicologic assessment of genetically modified drought-resistant wheat T349 with GmDREB1. Zhonghua Yu Fang Yi Xue Za Zhi.

[22] Li Y, Zhang H, Zhang Q, Liu Q, Zhai H, Zhao N, He S (2019). An AP2/ERF gene, IbRAP2-12, from sweetpotato is involved in salt and drought tolerance in transgenic Arabidopsis. Plant Sci.

[23] Hong JP, Kim WT (2005). Isolation and functional characterization of the ca-DREBLP1 gene encoding a dehydration-responsive element binding-factor-like protein 1 in hot pepper (Capsicum annuum L. cv. Pukang). Planta.

[24] Cao B, Shu L, Li A (2019). Functional characterization of LkERF-B2 for improved salt tolerance ability in ***Arabidopsis thaliana***. 3 Biotech.

[25] Meng XQ, Liu SY, Dong TT, Li ZY, Ma DF, Pan SY, Zhu MK (2020). Identification, expression analysis, and functional characterization of salt stress-responsive genes of AP2/ERF transcription factors in sweetpotato. Crop Sci.

[26] Aukerman MJ, Sakai H (2003). Regulation of flowering time and floral organ identity by a MicroRNA and its APETALA2-like target genes. Plant Cell.

[27] Duran-Medina Y, Serwatowska J, Reyes-Olalde JI, de Folter S, Marsch-Martinez N (2017). The AP2/ERF transcription factor DRNL modulates gynoecium development and affects its response to Cytokinin. Front Plant Sci.

[28] Gao Y, Liu Y, Liang Y, Lu J, Jiang C, Fei Z, Jiang CZ, Ma C, Gao J (2019). Rosa hybrida RhERF1 and RhERF4 mediate ethylene- and auxin-regulated petal abscission by influencing pectin degradation. Plant J.

[29] Jiang F, Guo M, Yang F, Duncan K, Jackson D, Rafalski A, Wang S, Li B (2012). Mutations in an AP2 transcription factor-like gene affect internode length and leaf shape in maize. PLoS One.

[30] Horstman A, Li MF, Heidmann I, Weemen M, Chen BJ, Muino JM, Angenent GC, Boutilier K (2017). The BABY BOOM transcription factor activates the LEC1-ABI3-FUS3-LEC2 network to induce somatic embryogenesis. Plant Physiol.

[31] Xie Z, Nolan TM, Jiang H, Yin Y (2019). AP2/ERF transcription factor regulatory networks in hormone and abiotic stress responses in Arabidopsis. Front Plant Sci.

[32] Hu YX, Wang YH, Liu XF, Li JY (2004). Arabidopsis RAV1 is down-regulated by brassinosteroid and may act as a negative regulator during plant development. Cell Res.

[33] Alonso JM, Stepanova AN, Leisse TJ, Kim CJ, Chen HM, Shinn P, Stevenson DK, Zimmerman J, Barajas P, Cheuk R, Gadrinab C, Heller C, Jeske A, Koesema E, Meyers CC, Parker H, Prednis L, Ansari Y, Choy N, Deen H, Geralt M, Hazari N, Hom E, Karnes M, Mulholland C, Ndubaku R, Schmidt I, Guzman P, Aguilar-Henonin L, Schmid M, Weigel D, Carter DE, Marchand T, Risseeuw E, Brogden D, Zeko A, Crosby WL, Berry CC, Ecker JR (2003). Genome-wide insertional mutagenesis of Arabidopsis thaliana. Science.

[34] Kitomi Y, Ito H, Hobo T, Aya K, Kitano H, Inukai Y (2011). The auxin responsive AP2/ERF transcription factor CROWN ROOTLESS5 is involved in crown root initiation in rice through the induction of OsRR1, a type-a response regulator of cytokinin signaling. Plant J.

[35] Li CW, Su RC, Cheng CP, Sanjaya YSJ, Hsieh TH, Chao TC, Chan MT (2011). Tomato RAV transcription factor is a pivotal modulator involved in the AP2/EREBP-mediated defense pathway. Plant Physiol.

[36] Gu C, Guo ZH, Hao PP, Wang GM, Jin ZM, Zhang SL (2017). Multiple regulatory roles of AP2/ERF transcription factor in angiosperm. Bot Stud.

[37] Hou F, Du T, Qin Z, Xu T, Li A, Dong S, Ma D, Li Z, Wang Q, Zhang L (2021). Genome-wide in silico identification and expression analysis of beta-galactosidase family members in sweetpotato [***Ipomoea batatas*** (L.) Lam]. BMC Genomics.

[38] Bovell-Benjamin AC (2007). Sweet potato: a review of its past, present, and future role in human nutrition. Adv Food Nutr Res.

[39] Jin R, Jiang W, Yan M, Zhang A, Liu M, Zhao P, Chen X, Tang Z (2021). Genome-wide characterization and expression analysis of HAK K(+) transport family in Ipomoea. 3 Biotech.

[40] Jin R, Zhang A, Sun J, Chen X, Liu M, Zhao P, Jiang W, Tang Z (2021). Identification of shaker K(+) channel family members in sweetpotato and functional exploration of IbAKT1. Gene.

[41] Kim YH, Yang KS, Ryu SH, Kim KY, Song WK, Kwon SY, Lee HS, Bang JW, Kwak SS (2008). Molecular characterization of a cDNA encoding DRE-binding transcription factor from dehydration-treated fibrous roots of sweetpotato. Plant Physiol Biochem.

[42] Kim YH, Jeong JC, Park S, Lee HS, Kwak SS (2012). Molecular characterization of two ethylene response factor genes in sweetpotato that respond to stress and activate the expression of defense genes in tobacco leaves. J Plant Physiol.

[43] Jin R, Kim BH, Ji CY, Kim HS, Li HM, Ma DF, Kwak SS (2017). Overexpressing IbCBF3 increases low temperature and drought stress tolerance in transgenic sweetpotato. Plant Physiol Biochem.

[44] Yu Y, Kim HS, Ma PY, Jia ZD, Guo XD, Xie YZ, Kwak SS, Zhang P, Bian XF (2020). A novel ethylene-responsive factor IbERF4 from sweetpotato negatively regulates abiotic stress. Plant Biotechnology Reports.

[45] Patel RK, Jain M (2012). NGS QC toolkit: a toolkit for quality control of next generation sequencing data. PLoS One.

[46] Zhan J, Thakare D, Ma C, Lloyd A, Nixon NM, Arakaki AM, Burnett WJ, Logan KO, Wang D, Wang X, Drews GN, Yadegari R (2015). RNA sequencing of laser-capture microdissected compartments of the maize kernel identifies regulatory modules associated with endosperm cell differentiation. Plant Cell.

[47] Ji CY, Kim HS, Lee CJ, Kim SE, Lee HU, Nam SS, Li Q, Ma DF, Kwak SS (2020). Comparative transcriptome profiling of tuberous roots of two sweetpotato lines with contrasting low temperature tolerance during storage. Gene.

[48] Xu J, Duan XG, Yang J, Beeching JR, Zhang P (2013). Enhanced reactive oxygen species scavenging by overproduction of superoxide dismutase and catalase delays postharvest physiological deterioration of cassava storage roots. Plant Physiol.

[49] Licausi F, Giorgi FM, Zenoni S, Osti F, Pezzotti M, Perata P (2010). Genomic and transcriptomic analysis of the AP2/ERF superfamily in Vitis vinifera. BMC Genomics.

[50] Song XM, Li Y, Hou XL. Genome-wide analysis of the AP2/ERF transcription factor superfamily in Chinese cabbage (Brassica rapa ssp pekinensis). BMC Genomics. 2013;14(1). 10.1186/1471-2164-14-573. 10.1186/1471-2164-14-573 PMC3765354 23972083

[51] Liu MY, Sun WJ, Ma ZT, Zheng TR, Huang L, Wu Q, Zhao G, Tang ZZ, Bu TL, Li CL (2019). Genome-wide investigation of the AP2/ERF gene family in tartary buckwheat (Fagopyum Tataricum). BMC Plant Biol.

[52] Du HW, Huang M, Zhang ZX, Cheng SY (2014). Genome-wide analysis of the AP2/ERF gene family in maize waterlogging stress response. Euphytica.

[53] Zhang CH, Shangguan LF, Ma RJ, Sun X, Tao R, Guo L, Korir NK, Yu ML (2012). Genome-wide analysis of the AP2/ERF superfamily in peach (Prunus persica). Genet Mol Res.

[54] Lata C, Mishra AK, Muthamilarasan M, Bonthala VS, Khan Y, Prasad M (2014). Genome-wide investigation and expression profiling of AP2/ERF transcription factor superfamily in foxtail millet (*Setaria italica* L.). PLoS One.

[55] Shu YJ, Liu Y, Zhang J, Song LL, Guo CH. Genome-wide analysis of the AP2/ERF superfamily genes and their responses to abiotic stress in Medicago truncatula. Front Plant Sci. 2016;6. 10.3389/fpls.2015.01247. 10.3389/fpls.2015.01247 PMC4717309 26834762

[56] Zhao T, Xia H, Liu JY, Ma FW (2014). The gene family of dehydration responsive element-binding transcription factors in grape (Vitis vinifera): genome-wide identification and analysis, expression profiles, and involvement in abiotic stress resistance. Mol Biol Rep.

[57] Guo BJ, Wei YF, Xu RB, Lin S, Luan HY, Lv C, Zhang XZ, Song XY, Xu RG: Genome-Wide Analysis of APETALA2/Ethylene-Responsive Factor (AP2/ERF) Gene Family in Barley (*Hordeum vulgare* L.). PLoS One 2016, 11, 9, DOI: 10.1371/journal.pone.0161322. 10.1371/journal.pone.0161322 PMC5012588 27598245

[58] Karanja BK, Xu L, Wang Y, Tang MJ, Muleke EM, Dong JH, Liu LW (2019). Genome-wide characterization of the AP2/ERF gene family in radish (*Raphanus sativus* L.): Unveiling evolution and patterns in response to abiotic stresses. Gene.

[59] Li H, Wang Y, Wu M, Li LH, Li C, Han ZP, et al. Genome-wide identification of AP2/ERF transcription factors in cauliflower and expression profiling of the ERF family under salt and drought stresses. Front Plant Sci. 2017;8. 10.3389/fpls.2017.00946. 10.3389/fpls.2017.00946 PMC5462956 28642765

[60] Tang YH, Qin SS, Guo YL, Chen YB, Wu PZ, Chen YP, Li MR, Jiang HW, Wu GJ: Genome-Wide Analysis of the AP2/ERF Gene Family in Physic Nut and Overexpression of the JcERF011 Gene in Rice Increased Its Sensitivity to Salinity Stress. PLoS One 2016, 11, 3, DOI: 10.1371/journal.pone.0150879. 10.1371/journal.pone.0150879 PMC4778941 26943337

[61] Zhang Z, Li XG. Genome-wide identification of AP2/ERF superfamily genes and their expression during fruit ripening of Chinese jujube. Sci Rep. 2018;8(1). 10.1038/s41598-018-33744-w. 10.1038/s41598-018-33744-w PMC6199273 30353116

[62] Liu LS, White MJ, MacRae TH (1999). Transcription factors and their genes in higher plants - functional domains, evolution and regulation. Eur J Biochem.

[63] Franco-Zorrilla JM, Lopez-Vidriero I, Carrasco JL, Godoy M, Vera P, Solano R (2014). DNA-binding specificities of plant transcription factors and their potential to define target genes. Proc Natl Acad Sci U S A.

[64] Ikeda M, Ohme-Takagi M (2009). A novel group of transcriptional repressors in Arabidopsis. Plant Cell Physiol.

[65] Kagale S, Rozwadowski K (2011). EAR motif-mediated transcriptional repression in plants: an underlying mechanism for epigenetic regulation of gene expression. Epigenetics.

[66] Tiwari SB, Belachew A, Ma SF, Young M, Ade J, Shen Y, Marion CM, Holtan HE, Bailey A, Stone JK, Edwards L, Wallace AD, Canales RD, Adam L, Ratcliffe OJ, Repetti PP (2012). The EDLL motif: a potent plant transcriptional activation domain from AP2/ERF transcription factors. Plant J.

[67] Marchler-Bauer A, Bo Y, Han L, He J, Lanczycki CJ, Lu S, Chitsaz F, Derbyshire MK, Geer RC, Gonzales NR, Gwadz M, Hurwitz DI, Lu F, Marchler GH, Song JS, Thanki N, Wang Z, Yamashita RA, Zhang D, Zheng C, Geer LY, Bryant SH (2017). CDD/SPARCLE: functional classification of proteins via subfamily domain architectures. Nucleic Acids Res.

[68] Li MY, Xu ZS, Huang Y, Tian C, Wang F, Xiong AS (2015). Genome-wide analysis of AP2/ERF transcription factors in carrot (Daucus carota L.) reveals evolution and expression profiles under abiotic stress. Mol Gen Genomics.

[69] Mizoi J, Shinozaki K, Yamaguchi-Shinozaki K (2012). AP2/ERF family transcription factors in plant abiotic stress responses. Biochim Et Biophys Acta.

[70] Zhu D, Wu Z, Cao G, Li J, Wei J, Tsuge T, Gu H, Aoyama T, Qu LJ (2014). TRANSLUCENT GREEN, an ERF family transcription factor, controls water balance in Arabidopsis by activating the expression of aquaporin genes. Mol Plant.

[71] Cheng MC, Hsieh EJ, Chen JH, Chen HY, Lin TP (2012). Arabidopsis RGLG2, functioning as a RING E3 ligase, interacts with AtERF53 and negatively regulates the plant drought stress response. Plant Physiol.

[72] Cheng MC, Liao PM, Kuo WW, Lin TP (2013). The Arabidopsis ETHYLENE RESPONSE FACTOR1 regulates abiotic stress-responsive gene expression by binding to different cis-acting elements in response to different stress signals. Plant Physiol.

[73] Jeon J, Cho C, Lee MR, Van Binh N, Kim J (2016). CYTOKININ RESPONSE FACTOR2 (CRF2) and CRF3 regulate lateral root development in response to cold stress in Arabidopsis. Plant Cell.

[74] Shigyo M, Hasebe M, Ito M (2006). Molecular evolution of the AP2 subfamily. Gene.

[75] Lata C, Prasad M (2011). Role of DREBs in regulation of abiotic stress responses in plants. J Exp Bot.

[76] Barnaby JY, Kim J, Devi MJ, Fleisher DH, Tucker ML, Reddy VR, et al. Varying Atmospheric CO2 Mediates the Cold-Induced CBF-Dependent Signaling Pathway and Freezing Tolerance in Arabidopsis. Int J Mol Sci. 2020;21(20). 10.3390/ijms21207616. 10.3390/ijms21207616 PMC7593905 33076265

[77] Krishnaswamy S, Verma S, Rahman MH, Kav NN (2011). Functional characterization of four APETALA2-family genes (RAP2.6, RAP2.6L, DREB19 and DREB26) in Arabidopsis. Plant Mol Biol.

[78] Dietz KJ, Vogel MO, Viehhauser A (2010). AP2/EREBP transcription factors are part of gene regulatory networks and integrate metabolic, hormonal and environmental signals in stress acclimation and retrograde signalling. Protoplasma.

