# Correction to: Genome-wide identification, phylogeny and expression analysis of AP2/ERF transcription factors family in sweet potato

**DOI:** 10.1186/s12864-022-08355-5

**Published:** 2022-02-16

**Authors:** Shutao He, Xiaomeng Hao, Shuli He, Xiaoge Hao, Peng Zhang, Xiaonan Chen

**Affiliations:** 1 State Key Laboratory of Cell Biology, Shanghai Institute of Biochemistry and Cell Biology, CAS Center for Excellence in Molecular Cell Science, Chinese Academy of Sciences, Shanghai, 200031 China; 2 National Key Laboratory of Plant Molecular Genetics, CAS Center for Excellence in Molecular Plant Sciences, Institute of Plant Physiology and Ecology, Chinese Academy of Sciences, Shanghai, 200032 China; 3 University of Chinese Academy of Sciences, Beijing, 100049 China; 4 Jining College Affiliated Senior High School, Jining, 272004 China; 5 Tsinghua University, Beijing, 100084 China; 6 Beihang University, Beijing, 100191 China

**Correction to: BMC Genomics 22, 748 (2021)**

**https://doi.org/10.1186/s12864-021-08043-w**

Following publication of the original article [1], it was reported that the authorship was missing the author Peng Zhang. The correct authorship list and updated Authorship contribution and Acknowledgements declarations are included in this Correction article.

The original article [1] has been corrected.

**Acknowledgements**

We are grateful to Dr. Luonan Chen, Dr. Chuanchao Zhang and Dr. Qianqian Shi for their constructive suggestion on data analysis. This work was partially supported by National Key R&D Program of China (2018YFD1000705). We appreciate the editor and reviewers for critically evaluating this manuscript and providing constructive comments for its improvement.

**Authors’ contributions**

Shutao He, Peng Zhang and Xiaonan Chen designed and conceived this project; Shutao He, Xiaomeng Hao, Xiaoge Hao and Shuli He performed the analysis and experiments; Shutao He and Xiaomeng Hao prepared the figure and drafted the manuscript; Xiaonan Chen, Xiaoge Hao and Shuli He analyzed the data and revised the manuscript with input from the other authors. All authors contributed to and approved the final manuscript.
