# Supplementary material for: Genome-wide identification, phylogeny and expression analysis of AP2/ERF transcription factors family in sweet potato
Source: BMC Genomics. 2021 Oct 16;22:748. doi: 10.1186/s12864-021-08043-w (PMC8520649; doi:10.1186/s12864-021-08043-w)
Supplement: Supplementary file 2 — Additional file 2: Fig. S1. Phylogenetic relationships and conserved domains in IbAP2/ERF proteins from sweet potato. [file 12864_2021_8043_MOESM2_ESM.pdf]

Figure S1.

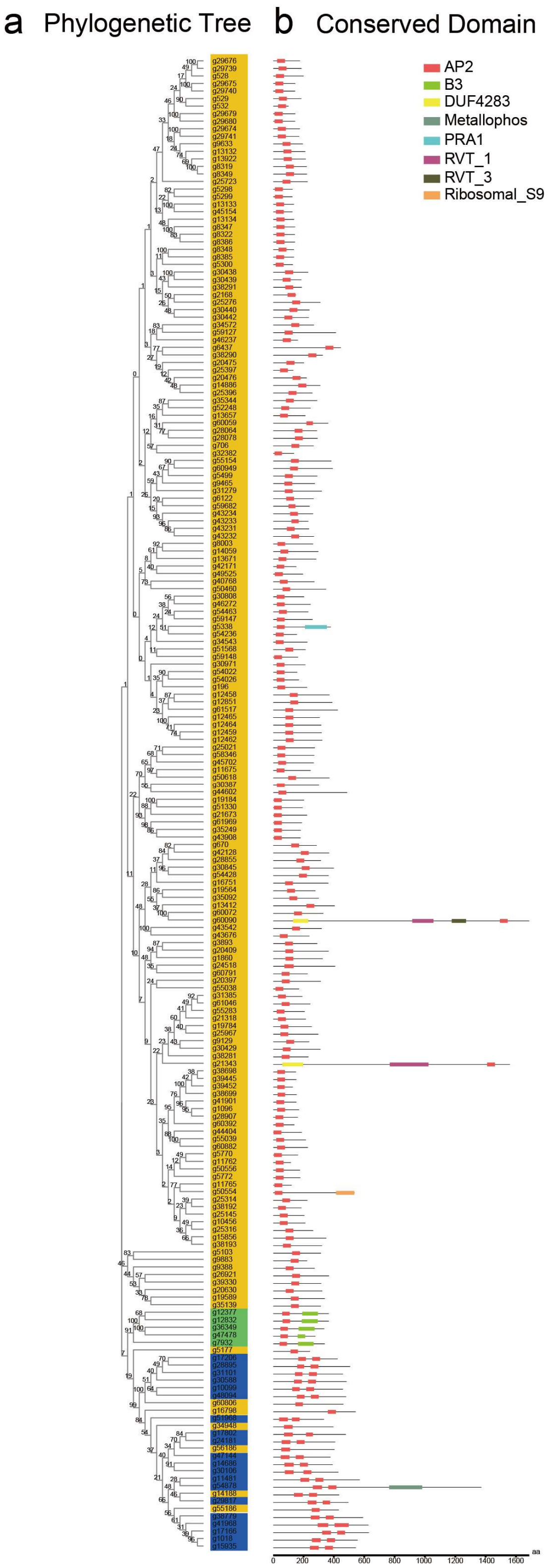

Figure S1. Phylogenetic relationships and conserved domains in IbAP2/ERF proteins from sweet potato. (a) The phylogenetic tree was constructed of IbAP2/ERF proteins in sweet potato. Members of *ERF*, *AP2* and *RAV* subfamily were filled in yellow, blue and green respectively. (b) Conserved domains of sweet potato IbAP2/ERF proteins. The AP2 domain is filled with red boxes and B3 domain with green boxes.
